# A novel mechanism of hippocampal LTD involving muscarinic receptor-triggered interactions between AMPARs, GRIP and liprin-α

**DOI:** 10.1186/1756-6606-2-18

**Published:** 2009-06-17

**Authors:** Bryony A Dickinson, Jihoon Jo, Heon Seok, Gi Hoon Son, Daniel J Whitcomb, Ceri H Davies, Morgan Sheng, Graham L Collingridge, Kwangwook Cho

**Affiliations:** 1 Henry Wellcome Laboratories for Integrative Neuroscience and Endocrinology (LINE), Faculty of Medicine and Dentistry, University of Bristol, Whitson Street, Bristol BS1 3NY, UK; 2 Department of Anatomy, University of Bristol, MRC Centre for Synaptic Plasticity, University Walk, Bristol BS8 1TD, UK; 3 Neurosciences CEDD, GlaxoSmithKline, New Frontiers Science Park North, Third Avenue, Harlow, Essex CM19 5AW, UK; 4 Department of Brain and Cognitive Sciences, Picower Institute for Learning and Memory, Massachusetts Institute of Technology, Cambridge, Massachusetts 02139, USA

## Abstract

**Background:**

Long-term depression (LTD) in the hippocampus can be induced by activation of different types of G-protein coupled receptors, in particular metabotropic glutamate receptors (mGluRs) and muscarinic acethycholine receptors (mAChRs). Since mGluRs and mAChRs activate the same G-proteins and isoforms of phospholipase C (PLC), it would be expected that these two forms of LTD utilise the same molecular mechanisms. However, we find a distinct mechanism of LTD involving GRIP and liprin-α.

**Results:**

Whilst both forms of LTD require activation of tyrosine phosphatases and involve internalisation of AMPARs, they use different molecular interactions. Specifically, mAChR-LTD, but not mGluR-LTD, is blocked by peptides that inhibit the binding of GRIP to the AMPA receptor subunit GluA2 and the binding of GRIP to liprin-α. Thus, different receptors that utilise the same G-proteins can regulate AMPAR trafficking and synaptic efficacy via distinct molecular mechanisms.

**Conclusion:**

Our results suggest that mAChR-LTD selectively involves interactions between GRIP and liprin-α. These data indicate a novel mechanism of synaptic plasticity in which activation of M1 receptors results in AMPAR endocytosis, via a mechanism involving interactions between GluA2, GRIP and liprin-α.

## Background

Cholinergic neurotransmission in the brain has a critical role in cognition [1-4]. In particular, inhibition of muscarinic receptors produces pronounced amnesia and loss of cholinergic innervation is an early feature of Alzheimer's disease (AD) [5-8]. As a result, the primary treatment for the cognitive deficits in AD is cholinesterase inhibitors, used to increase the amount of ACh available to activate neurons. In addition, there is increasing interest in the use of agents that specifically activate muscarinic AChRs (mAChRs) for the treatment of both AD [9-11] and schizophrenia [12]. It is therefore extremely important to understand how ACh regulates synaptic function, particularly that which is relevant to learning and memory.

In this context, activation of mAChRs using carbachol (CCh) induces LTD of excitatory synaptic transmission in various brain regions, including the visual cortex [13-15], perirhinal cortex [16,17] and hippocampus [13,18-21]. However, the molecular mechanisms of mAChR-dependent LTD are poorly understood. In the present study we have therefore investigated the mechanisms involved in CCh-induced LTD (mAChR-LTD) in the hippocampus of adult rats. We find that activation of M1 receptors results in an LTD that is dependent on the activity of protein tyrosine phosphatases (PTPs), but is independent of Ca^2+^, PKC, serine/threonine protein phosphatases and protein synthesis. In all of these respects, this form of LTD is the same as that induced by activation of mGlu5 receptors in hippocampal slices obtained from adult animals [22,23]. However, to our surprise, we found that mAChR-LTD, but not mGluR-LTD, involves interactions between GRIP and the AMPAR subunit GluA2 (IUPHAR nomenclature for subunits previously known as GluR2 or GluRB; see [24]). Furthermore, mAChR-LTD also selectively involves interactions between GRIP and liprin-α. These data indicate a novel mechanism of synaptic plasticity in which activation of M1 receptors results in AMPAR endocytosis, via a mechanism involving interactions between GluA2, GRIP and liprin-α.

### Results

#### Carbachol induces an NMDAR-independent form of LTD in the CA1 area

**Figure 1:**
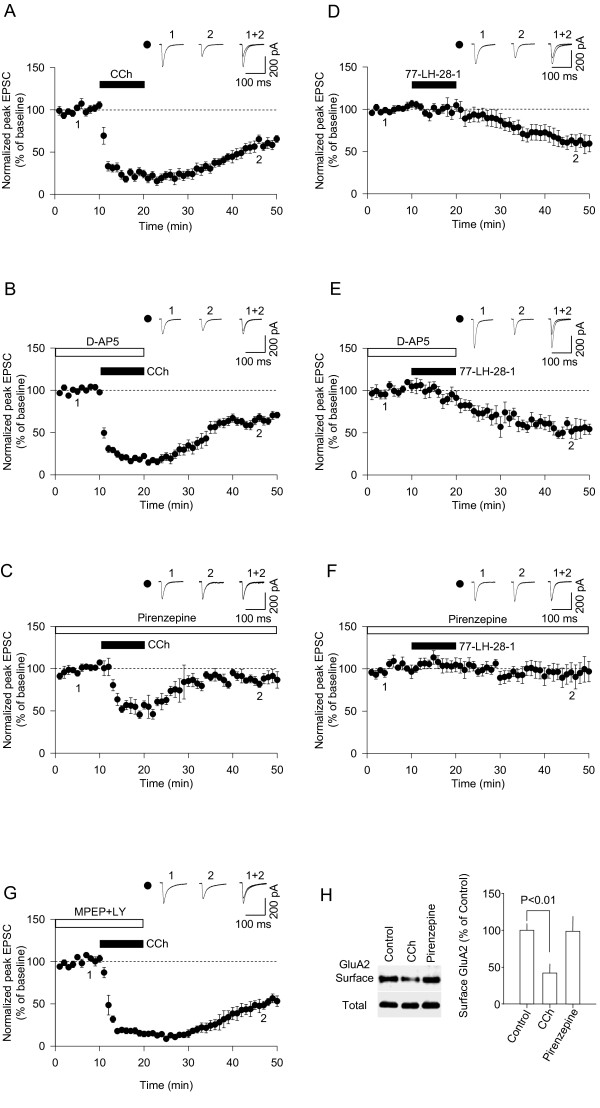
**Properties of CCh-induced LTD in the CA1 region of the hippocampus**. (A) A pooled data (n = 8) of EPSC amplitude *vs *time to show that carbachol application (CCh, 50 μM, 10 min) induces mAChR-LTD. (B) D-AP5 (50 μM) has no effect on mAChR-LTD (n = 9). (C) mAChR-LTD was prevented by bath application of an M1 mAChR antagonist, pirenzepine (0.5 μM, n = 5). (D) The M1 specific agonist, 77-LH-28-1, induces LTD (n = 6). (E) D-AP5 (50 μM) has no effect on LTD induced by 77-LH-28-1 (n = 5). (F) Pirenzepine (0.5 μM) prevents LTD induced by 77-LH-28-1 (n = 7). (G) Co-application of LY367385 (100 μM) and MPEP (50 μM) has no effect on mAChR-LTD (n = 6). (H) An example of biotinylation from hippocampal slices. Pooled data (n = 4) shows that CCh induces internalisation of GluA2. Error bars represent s.e.m.

Bath application of carbachol (CCh; 50 μM, 10 min) resulted in LTD of synaptic transmission in the CA1 region of the hippocampus in 4–5 week old rats (56% ± 7% of baseline, quantified 30 min following washout of CCh; n = 8) (Figure 1A). A similar LTD was induced when CCh was applied in the presence of an NMDAR antagonist, D-AP5 (58% ± 5%, n = 9) (Figure 1B), demonstrating that this is an NMDAR-independent form of synaptic plasticity. The AChR-LTD involved activation of M1 receptors, since it was significantly reduced by pirenzepine (0.5 μM) (88% ± 7%, n = 5 [p < 0.05 vs control LTD]) (Figure 1C). In addition, the M1 selective agonist 77-LH-28-1 (10 μM) induced a slow-onset LTD (60% ± 8%, n = 6) (Figure 1D) that was also resistant to treatment with D-AP5 (61% ± 7%, n = 5) (Figure 1E) and was blocked by pirenzepine (93% ± 11%, n = 7 [p < 0.05 vs control]) (Figure 1F). The CCh-induced LTD resembles that induced by group I mGluRs and so could conceivably be due to CCh facilitation of endogenous L-glutamate actions on group I mGluRs. However, this was not the case, since CCh-induced LTD was completely resistant to inhibitors of group I mGluRs (58% ± 13%, n = 6) (Figure 1G).

To investigate the expression mechanism of this mAChR-LTD, we performed surface biotinylation assays using hippocampal slices. Hippocampal slices were treated with CCh, in the presence or absence of pirenzepine, and the cell surface and total expression level of GluA2 subunits was compared. CCh induced a substantial internalisation of GluA2 subunits (Figure 1H), consistent with a mechanism that involves the internalisation of AMPARs [20].

### Signalling mechanisms involved in mAChR-LTD

**Figure 2:**
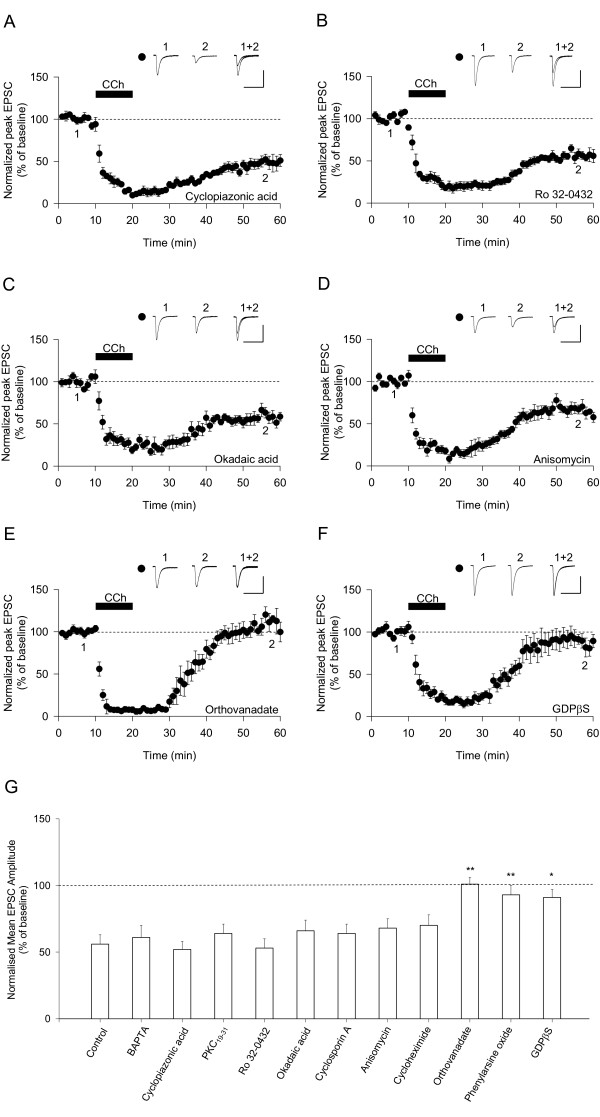
**Signalling mechanisms involved in mAChR-LTD**. (A) Cyclopiazonic acid (2 μM) has no effect on mAChR-LTD (n = 6). (B) Ro 32-0432 (10 μM) has no effect on mAChR-LTD (n = 6). (C) Okadaic acid (100 nM) has no effect on mAChR-LTD (n = 5). (D) Anisomycin (20 μM) has no effect on mAChR-LTD (n = 7). (E) Orthovanadate (100 μM) prevents the induction of mAChR-LTD (n = 5). (F) GDPβS (1 mM) blocks the induction of mAChR-LTD (n = 6). (G) A summary of results from control (n = 8), BAPTA (n = 9), cyclopiazonic acid (n = 6), PKC19–31 (n = 9), Ro 32-0432 (n = 6), okadaic acid (n = 5), cyclosporin A (n = 7), anisomycin (n = 7), cycloheximide (n = 7), orthovanadate (n = 5), phenylarsine oxide (n = 7) and GDPβS (n = 6), experiments. * P < 0.05 vs control ** P < 0.01 vs control.

M1 receptors conventionally signal via IP_3_-induced Ca^2+ ^release from intracellular stores and/or activation of PKC [25-28]. However, intracellular infusion of cyclopiazonic acid (CPA, 2 μM), which depletes Ca^2+ ^stores, had no effect on mAChR-LTD (52% ± 6%, n = 6) (Figure 2A). Similarly, postsynaptic infusion of either the PKC inhibitor Ro 32-0432 (10 μM, 53% ± 7%, n = 6) (Figure 2B) or the inhibitory peptide PKC_19–31 _(10 μM; 64% ± 7%, n = 9) (data not shown) had no effect on mAChR-LTD. Therefore, it would seem that mAChR-LTD involves an unconventional signalling mechanism. An alternative possibility is that mAChR-LTD involves a different Ca^2+^-dependent process, since most forms of synaptic plasticity are Ca^2+^-dependent [29]. However, postsynaptic infusion of BAPTA (10 mM) had no effect on mAChR-LTD (61% ± 9%, n = 9) (data not shown).

The serine/threonine protein phosphatases PP1 and PP2B (calcineurin) are required for NMDAR-dependent LTD [30,31]. To determine whether these enzymes are important for mAChR-LTD we included either okadaic acid or cyclosporin-A in the whole-cell solution. However, neither okadaic acid (100 nM, 66% ± 8%, n = 5) (Figure 2C) nor cyclosporin-A (10 μM, 64% ± 7%, n = 7) (data not shown) had any effect on mAChR-LTD. Another candidate mechanism for mAChR-LTD involves protein synthesis [16,20]. Therefore it was surprising to find that neither of the protein translation inhibitors anisomycin (20 μM, 68% ± 7%, n = 7) (Figure 2D) nor cycloheximide (80 μM, 70% ± 8%, n = 7) (data not shown) had any significant effect on mAChR-LTD.

These negative findings are reminiscent of mGluR-LTD in the CA1 region of the hippocampus of adult rats [22,23]. Since this latter form of LTD is blocked by broad spectrum PTP inhibitors, we tested orthovanadate and phenylarsine oxide (PAO) on mAChR-LTD. Both orthovanadate (100 μM, 101% ± 5%, n = 5) (Figure 2E) and PAO (1.5 μM, 93% ± 7%, n = 7) (data not shown) blocked mAChR-LTD. Finally, we tested whether, like mGluR-LTD [32], mAChR-LTD requires activation of G-proteins or whether it operates in a G-protein independent manner (see [33]). Postsynaptic inclusion of guanosine-5'-*O*-(2-thiodiphosphate) (GDPβS) inhibited mAChR-LTD (1 mM, 91% ± 6%, n = 6) (Figure 2F), confirming that a G-protein signalling mechanism is involved. These results, which are summarised in Figure 2G, show that mAChR-LTD involves very similar signalling mechanisms to that previously described for mGluR-LTD in adult hippocampus [22,23,32].

### An interaction between GluA2 and GRIP is necessary for mAChR-LTD

**Figure 3:**
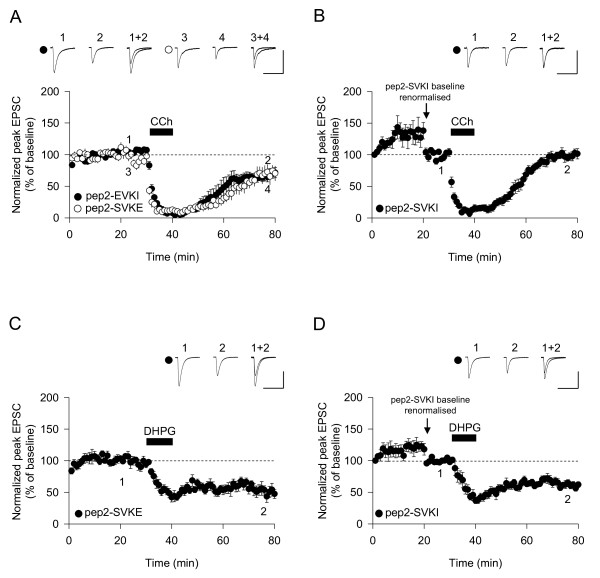
**Interactions between GluA2 and GRIP, but not PICK1, are required for mAChR-LTD**. (A) Neither pep2-SVKE (n = 6) nor pep2-EVKI (n = 9) has any effect on mAChR-LTD. (B) Pep2-SVKI prevents mAChR-LTD (n = 8). (C) Pep2-SVKE (n = 6) has no effect on mGluR-LTD. (D) Pep2-SVKI (n = 6) has no effect on mGluR-LTD.

How activation of PTPs results in LTD is not known, but the finding that both mGluR-LTD and mAChR-LTD involve internalisation of AMPARs suggests that proteins that interact with these receptors might be involved. In the ventral tegmental area (VTA) it has been shown that blocking the interaction between GluA2 and PICK1, with the peptide inhibitor pep2-EVKI (YNVYGIEEVKI) [34,35], prevents mGluR-LTD [36]. In addition, blocking GluA2 interactions with PICK1 also prevents mGluR-LTD in the cerebellum [37]. We therefore included pep2-EVKI (100 μM) in the whole-cell solution and compared its effects with that of a control peptide, pep2-SVKE (100 μM), which has no effect on GluA2-PDZ interactions [34,35]. We found that neither pep2-EVKI (64% ± 6%, n = 9) (Figure 3A) nor pep2-SVKE (67% ± 13%, n = 6) (Figure 3A) had any effect on mAChR-LTD. We therefore tested pep2-SVKI (YNVYGIESVKI), which in addition to blocking PICK1 interactions with GluA2 also blocks GRIP (ABP) interactions with this subunit [34,35]. We found that pep2-SVKI (100 μM) caused a characteristic run-up in synaptic transmission [35] and, most surprisingly, blocked mAChR-LTD (97% ± 9%, n = 8) (Figure 3B). These interfering peptide experiments suggest that GRIP rather than PICK1 is involved in mAChR-LTD.

Given the identical signalling cascades triggered by both M1 and mGlu5 receptors it was natural to assume that pep2-SVKI should also block DHPG-LTD. Remarkably, however, it did not. Thus, the levels of LTD induced in cells loaded with pep2-SVKE (54% ± 8%, n = 6) (Figure 3C) and pep2-SVKI (61% ± 4%, n = 6) (Figure 3D) were not significantly different. These results demonstrate a divergence at the level of AMPAR trafficking between these two forms of LTD, despite the similarity in signal transduction mechanisms.

### GRIP1-Liprin-α association has a critical role in mAChR-LTD

We sought an explanation how GRIP might be involved in mAChR-LTD. In this context, an association between GRIP and liprin-α is important for synaptic targeting of AMPA receptors [38,39]. Liprin-α directly interacts with GRIP through its PDZ6 domain [38] and it also recruits leukocyte common antigen-related receptor (LAR) to GRIP [39]. LAR is a PTP that is known to be involved in axonal guidance and neuronal development including cholinergic network formation [40,41]. Therefore we determined whether the GRIP-liprin-α association has a role in mAChR-LTD.

**Figure 4:**
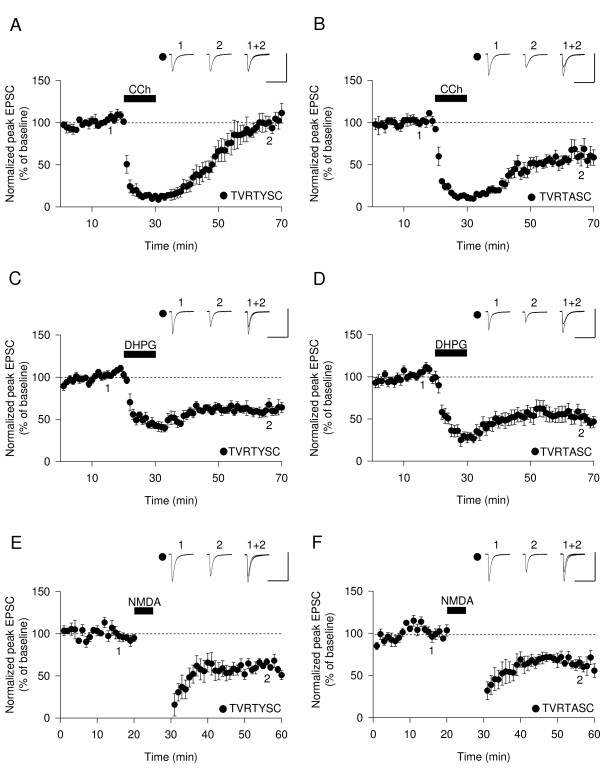
**Interaction between liprin α and GRIP is required for mAChR-LTD**. (A) Intracellular infusion of the C terminal fragment of liprin α (TVRTYSC) prevents induction of mAChR-LTD (n = 6). (B) A control peptide (TVRTASC) has no affect on mAChR-LTD (n = 5). (C) TVRTYSC (n = 7) has no effect on mGluR-LTD. (D) TVRTASC (n = 8) has no effect on mGluR-LTD. (E) TVRTYSC (n = 6) has no effect on NMDAR-LTD. (F) TVRTASC (n = 6) has no effect on NMDAR-LTD.

**Figure 5:**
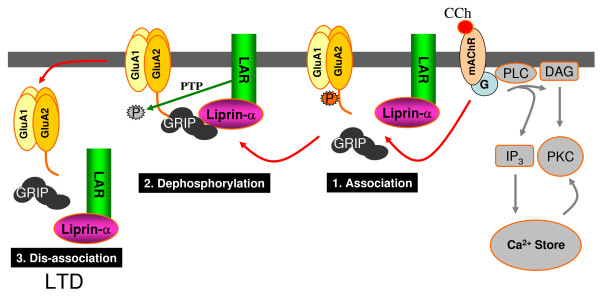
**A novel mechanism of LTD involving liprin-α and LAR**. Activation of mAChRs leads to a G-protein dependent mAChR-LTD that does not involve the canonical pathway (IP3 and PKC). The data can most simply be explained by GRIP acting as a targeting molecule that brings LAR and the GluA2 subunit of AMPARs into contact. This then enables LAR to dephosphorlyate a tyrosine residue (such as YGIESVKI on GluA2) which initiates the removal of the AMPAR from the synapse.

To investigate the potential role of the GRIP-liprin-α association in mAChR-LTD we included a peptide in the patch pipette (TVRTYSC) (100 μM) that corresponds to the C-terminal region of liprin-α, which is the interaction site with the PDZ6 domain of GRIP [38]. We interleaved these experiments with a control peptide (TVRTASC) (100 μM), which is unable to bind to GRIP due to an alanine substitution for tyrosine in the -2 position [38]. Whilst the C-terminal fragment blocked mAChR-LTD (98% ± 9%, n = 6) (Figure 4A) the control peptide did not (59% ± 7%, n = 5) (Figure 4B). To investigate whether the GRIP-liprin-α interaction is specifically required for mAChR-LTD we also investigated both mGluR-LTD and NMDAR-LTD. Interestingly, neither the active (60% ± 8%, n = 7) (Figure 4C) nor control (59% ± 9%, n = 8) (Figure 4D) peptides had any effect on DHPG-LTD. Similarly, the active (62% ± 3%, n = 6) (Figure 4E) and control (62% ± 6%, n = 6) (Figure 4F) peptides were also without effect on NMDA-induced LTD. These data indicate a specific role for the interaction between GRIP and liprin-α in the induction of mAChR-LTD (see Figure 5).

## Discussion

In the present study we have investigated a form of LTD involving muscarinic activation that leads to tyrosine dephosphorylation and the removal of AMPARs from the cell surface. Novel aspects of this work include the observations that the process involves interactions between the GluA2 subunit and GRIP and between GRIP and liprin-α, a protein that targets the PTP, LAR to GRIP. Remarkably, LTD induced by group I mGluRs does not utilise this same set of protein interactions, despite being triggered by activation of the same class of G protein and involving similar signal transduction mechanisms. These results point to a hitherto unexpected and remarkable degree of specificity in the protein: protein interactions that are involved in very similar forms of synaptic plasticity.

### Receptor mechanisms involved in CCh-LTD

LTD induced by the activation of muscarinic ACh receptors has been described in several brain regions, in particular the hippocampus [13,19-21,42], visual cortex [13-15,43] and perirhinal cortex [16,17]. In some instances the LTD is dependent on the activation of NMDARs [13,19,43] whilst in others it is not [14,16,20]. It is established that stimulation of muscarinic receptors can facilitate the activation of NMDARs [44-46]. It is likely therefore that the LTD that is sensitive to NMDAR blockade involves a muscarinic modulation of NMDAR-dependent LTD. In contrast, the LTD that is insensitive to NMDAR blockade is an independent form of LTD. In the present study the LTD that we have studied was of the latter variety since it was unaffected by D-AP5. This LTD resembles that induced by other Gq coupled receptors, such as the extensively characterised LTD induced through the activation of group I mGluRs by DHPG [e.g. [47-52]]. Other Gq coupled receptors can also induce LTD [21,53] suggesting that these neurotransmitters converge at the level of the G-protein with respect to their involvement in LTD. Consistent with previous work, CCh-induced LTD is mediated via activation of M1 receptors [14,19] whilst the initial depression requires activation of a different muscarinic subtype [19,42].

### Signalling mechanisms involved in mAChR-LTD

We tested a number of different inhibitors of cell signalling pathways to elucidate the pathways that lead from mAChR activation to AMPAR internalisation. In many cases we obtained negative results but this is not due to ineffective inhibition of the target compound. Not only were the inhibitors applied directly to the postsynaptic cell via the patch pipette, at concentrations known to be effective in other experiments, but in most cases we found, during parallel experiments, that the same compounds were effective on other forms of synaptic plasticity (e.g. [54]).

Compared to DHPG-LTD very little is known about the downstream signalling during mAChR-LTD. Classically, stimulation of M1 receptors leads to activation of PKC and the release of Ca^2+ ^from intracellular stores. However, we found no evidence that either limb of this pathway was involved in mAChR-LTD. The lack of effects of PKC inhibitors agree with previous studies of LTD induced by carbachol [14] and DHPG [55,56]. The effect of interfering with Ca^2+ ^stores is less clear, since a partial inhibition by CPA of CCh-LTD was observed in perirhinal cortex [16]. This might reflect a difference in brain region. In the present study, the LTD studied was also unaffected by BAPTA. This insensitivity to the chelation of intracellular Ca^2+ ^has also been reported for DHPG-LTD [57], and suggests that the signalling pathways involved in these Gq-dependent forms of synaptic plasticity can be Ca^2+^-independent. Previous work has implicated protein synthesis in mAChR-LTD. In two of these studies the effect of protein translation inhibitors were apparent rapidly but were only partially effective [16,20] whilst in another study these same inhibitors only affected mAChR-LTD after a delay of more than an hour [14]. In agreement with the latter report, we found no effect of protein translation inhibitors on mAChR-LTD during the duration of our experiments. A similar dichotomy has been reported with mGluR-LTD, with reports of both protein synthesis dependence [49] and independence [23], for reasons that are not clear. In terms of treatments that were effective, we did find that inhibition of PTPs completely prevented the induction of mAChR-LTD. This observation, together with the insensitivity to a serine/threonine protein phosphatase, again highlights similarities between mAChR-LTD and mGluR-LTD [22,23]. In summary, we can conclude that activation of M1 receptors results in the loss of surface AMPARs and the generation of LTD via a Ca^2+^-independent signalling cascade that involves one or more types of PTP.

### A role for GRIP in mAChR-LTD

Our study has demonstrated that mAChR-LTD induced by carbachol application is dependent on the internalisation of GluA2-containing AMPA receptors (see also [20]). A number of studies have shown that the induction of various forms of LTD involves phosphorylation and dephosphorylation events, which regulate interactions of PDZ domain proteins with AMPA receptors and induce AMPA receptor mobilisation (see, [58]). In particular, endocytosis of GluA2-containing AMPA receptors has previously been suggested to involve the PICK1-GluA2 interaction and a dependency upon PKC phosphorylation of S880 on the GluA2 subunit [59-62]. Indeed, there is considerable evidence for a role of PICK1 in mGluR-LTD in a variety of brain regions, including the cerebellum [37,63,64], VTA [65] and perirhinal cortex [54]. Surprisingly, therefore, we obtained no evidence for a role of PICK1 in mAChR-LTD in the hippocampus. This observation suggests that despite coupling to the same G-proteins and utilising similar signal transduction methods, mGluR-LTD and mAChR-LTD exploit different mechanisms at the level of AMPAR trafficking.

Whilst we found no evidence for a role of PICK1 in mAChR-LTD, we did find evidence of an essential role for GRIP. Although GRIP, and the related protein ABP, are established as important interactors with AMPARs [35,59,66,67] their precise roles are not known. For example, GRIP has been implicated in the stabilisation of AMPARs at synapses [59,61,62] and intracellular organelles [35,68] as well as in the sorting and transport of AMPARs [69,70]. Our results suggest that GRIP is also involved in the regulated synaptic removal of AMPARs. Specifically, blocking the interaction of GRIP with GluA2 prevents mAChR-LTD. This suggests that GRIP targets machinery to GluA2 that is involved in their synaptic removal. Remarkably, this effect is not part of a generalised LTD mechanism triggered by Gq-coupled receptor activation since mGluR-LTD was completely unaffected by blockade of the GluA2-GRIP interaction.

### A role for liprin-α in mAChR-LTD

An important interactor of GRIP is liprin-α (SYD2). This molecule binds to PDZ6 of GRIP and is involved in the surface expression and synaptic clustering of AMPARs [38]. Whether liprin-α is involved in the acute regulation of AMPAR synaptic expression, as occurs during LTP and LTD, is unknown. Our data, showing that a peptide capable of blocking the interaction of liprin-α with GRIP blocks mAChR-LTD, is consistent with the possibility that liprin-α plays a role in the rapid removal of AMPAR from synapses. Consistent with the unique role of GRIP in mAChR-LTD we found that the peptide designed to block the interaction between GRIP and liprin-α selectively blocks mAChR-LTD, having no effect on two other forms of LTD.

This raises the question as to how liprin-α might be functioning in mAChR-LTD. It is known that liprin-α binds the leukocyte common antigen-related (LAR) family receptor protein tyrosine phosphatase (LAR-RPTP). These PTPs are enriched at synapses and form complexes with GRIP and AMPARs [39], making them potential phosphatases involved in synaptic plasticity. Indeed, LAR-RPTPs could be the target of the broad spectrum PTP inhibitors that we have shown block mAChR-LTD. In contrast, since mGluR-LTD does not involve liprin-α, it is likely that it utilises a different PTP, such as STEP [71]. Conversely, NMDAR-LTD does not seem to involve PTPs of any kind, rather it involves serine/threonine protein phosphatases [31] and protein tyrosine kinases (PTKs) [72,73]. What is most clear from the present results is that there is a specific mechanism that is engaged for the regulation of synaptic AMPARs by the stimulation of muscarinic receptors, which is distinct from that employed by the activation of glutamate receptors. This might relate to the differences in the location of the glutamate receptors and muscarinic receptors that are activated by their respective neurotransmitters.

### Significance of the findings for cognition

The critical involvement of ACh in cognition is well established. It is likely that the ability of muscarinic receptor activation to modulate NMDAR-dependent synaptic plasticity and to induce synaptic plasticity in an NMDAR-independent manner are both important aspects of this function. Dissecting the relative roles of the cholinergic modulation of NMDAR-dependent synaptic plasticity and the cholinergic induction of LTD will be important challenges for the future. Interestingly, mGluR-LTD and mAChR-LTD are likely to be evoked under quite different conditions. The former requires strong activation of glutamatergic pathways and constitutes a form of homosynaptic plasticity. In contrast, mAChR-LTD can be induced with little or no activation of the glutamatergic system, and hence constitutes a form of heterosynaptic plasticity. In this way, cholinergic activation could simultaneously boost both NMDAR-dependent synaptic plasticity at strongly active inputs and depress transmission at inactive, or weakly active, inputs.

## Conclusion

We have identified a novel mechanism of synaptic plasticity that is specifically engaged during muscarinic receptor activation. This mechanism is not utilised by mGluR activation, demonstrating that different Gq-coupled receptors can affect AMPAR trafficking via distinct molecular mechanisms.

## Methods

### Electrophysiology

Hippocampal slices were obtained from 4–5 week old male Wistar rats. Animals were sacrificed by cervical dislocation in accordance with the UK Animals Scientific Procedures Act of 1986. The brains were quickly removed and transferred into ice-cold artificial cerebrospinal fluid (aCSF; bubbled with 95% O_2_/5% CO_2_) containing the following: (mM) NaCl, 124; KCl, 3; NaHCO_3_, 26; NaH_2_PO_4_, 1.25; CaCl_2_, 2; MgSO_4_, 1; D glucose, 10. Subsequently, a mid-sagittal cut was made in the brain and one hemisphere was placed back into the ice cold aCSF until it was required. Transverse hippocampal slices (400 μm) were prepared using either a vibratome (Leica, Nussloch, Germany) or a McIllwain tissue chopper (Mickle Laboratory Engineering Co. Ltd., Gomshall, UK). The slices were then submerged in aCSF (20°C–25°C) for at least 1 hour before recording. Slices were then transferred to the recording chamber and perfused with aCSF (28°C–30°C, flow rate 2~3 ml/min). Before recording, the CA3 region of the hippocampus was severed using a scalpel cut.

Whole-cell recordings were made from pyramidal cells in the CA1 region of the hippocampus (Axopatch 200 B amplifier, Molecular Devices, Sunnyvale, California). The patch pipette (resistance – 4–7 MΩ), pulled from borosilicate glass, was filled with a solution composed of (mM) CsMeSO_4_, 130; NaCl, 8; Mg-ATP, 4; Na-GTP, 0.3; EGTA, 0.5; HEPES 10; QX-314, 6 (280 mOsm [pH 7.2]). CA1 pyramidal neurons were voltage clamped at -70 mV and AMPA receptor-mediated synaptic currents were measured in the presence of picrotoxin (20 μM). Stimulating electrodes placed into the Schaffer collateral-commissural pathway, in the CA2 region, delivered stimuli at a frequency of 0.033 Hz. Series resistance and input resistance were monitored during the experiment and experimental data was not included if changes > 10% were seen.

In all experiments a baseline of at least 10 minutes was obtained before application of CCh or 77-LH-28-1. After drug application a washout period of 30–40 minutes was obtained. In experiments where pep2-SVKI, pep2-SVKE, pep2-EVKI, TVRTYSC and TVRTASC were incorporated into the pipette filling solution (Figure 3 and 4) a 20–30 minute baseline was obtained to ensure effective loading of the peptide and for stabilization of any effects on baseline transmission. The peptides, pep2-SVKI, pep2-SVKE and pep2-EVKI were purchased from Tocris (Bristol, UK) while TVRTYSC and TVRTASC were purchased from Peptide Protein Research LTD (Fareham, Hampshire, UK). BAPTA, cyclopiazonic acid, Ro 32-0432, PKC19-31, okadaic acid, cyclosporin A, anisomycin, cycloheximide, orthovanadate, phenylarsine oxide and GDPβS were added to the whole cell-patch filling solution. These chemicals were purchased from Calbiochem (California, U.S.A.). Picrotoxin, pirenzepine, and LY367385 were purchased from Tocris (Bristol, UK). Carbachol was purchased from SigmaAldrich (St Louis, U.S.A.). MPEP and D-AP5 was purchased from Ascent Scientific (Bristol, UK). These chemicals were made up as a stock solution and diluted to their final appropriate concentration in aCSF as required (indicated in Figures).

### Biotinylation

Surface expression of GluA2 was analysed with a commercial surface labelling kit according to the manufacturer's instructions (Thermo Fisher Scientific Inc., Rockford, IL, USA). Briefly, 400 μm thick hippocampal slices (6 slices for each lane) were incubated with aCSF containing 1 mg/ml sulfosuccinimidyl-6-(biotinamido) hexanoate for 45 min on ice, quenched by further incubation in aCSF containing 100 mM glycine, and followed by two washes in ice-cold Tris-buffered saline (50 mM Tris, pH 7.5, 150 mM NaCl). Crude cell lysates were prepared in modified RIPA buffer containing 50 mM Tris (pH 7.6), 150 mM NaCl, 0.5% Triton X-100, 0.5% sodium deoxycholate, 0.1% SDS, 5 mM NaF, 1 mM Na_3_VO_4 _and protease inhibitor cocktail (SigmaAldrich, St Louis, U.S.A.). Small aliquots of each lysates were kept for total GluA2 protein levels. The detergent-solubilized lysates were incubated with 50 μl of hydrated Neutravidin-Agarose beads for 4 h at 4°C to isolate biotinylated proteins. After the Neutravidin beads were washed four times with the RIPA buffer, bound proteins were eluted with SDS sample buffer by boiling for 5 min. Isolated biotinylated proteins and whole cell lysates were subsequently analyzed by western blotting with monoclonal anti-GluA2 (1:1,000; 556341, BD Bioscience, Frankin Lakes, NJ, USA). Immunoreactive bands were then probed with HRP-conjugated secondary antibody for 1 h and developed using the ECL detection system (Thermo Fisher Scientific Inc.). Equal loading of isolated surface proteins was confirmed based on silver-stained bands profiles on gels that were pre-run with small aliquots of samples. Optical densities of immunoreactivities were quantified using NIH ImageJ software (downloaded from ).

### Data Analysis

A sophisticated, free data acquisition and analysis package, the "LTP program" [74], was used to record the data, which had been filtered at 2 kHz and digitized at 10 kHz. During whole cell patch recording excitatory postsynaptic current (EPSC) amplitude, series resistance, DC current and input resistance were recorded. To graphically display the data, the amplitude of the EPSCs was normalized against baseline values and plotted against time. In the figures each data point represents the average of two raw data points. In each figure, data are shown as mean ± SEM. Where appropriate, the statistical significance of the data was established through use of a Student's t test, which was performed on EPSC amplitude measurements obtained during the 5 minutes before and between 25 and 30 minutes following washout of the muscarinic agonist.

## Competing interests

The authors declare that they have no competing interests.

## Authors' contributions

BAD conducted electrophysiology. JJ participated in the electrophysiology experiments. HS designed and participated in molecular experiments. GHS conducted molecular experiments. DJW participated in electrophysiology. CHD participated production of the M1 agonist. MS, GLC and KC conceived the original concept of this study and wrote the manuscript. KC supervised the entire project. All authors read and approved the final manuscript.
